# Improved culture enrichment broth for isolation of *Arcobacter-*like species from the marine environment

**DOI:** 10.1038/s41598-020-71442-8

**Published:** 2020-09-03

**Authors:** Faiz Ur Rahman, Karl B. Andree, Nuria Salas-Massó, Margarita Fernandez-Tejedor, Anna Sanjuan, Maria J. Figueras, M. Dolors Furones

**Affiliations:** 1 IRTA, Ctra. Poble Nou. Km 5.5, Sant Carles de la Ràpita, Spain; 2 Unit of Microbiology, Department of Basic Health Sciences, Faculty of Medicine and Health Sciences, IISPV, Universitat Rovira i Virgili, 34201 Reus, Spain

**Keywords:** Microbiology, Microbiology techniques

## Abstract

*Arcobacter*-like species are found associated with many matrices, including shellfish in marine environments. The culture media and conditions play a major role in the recovery of new *Arcobacter*-like species. This study was aimed to develop a culture media for isolation and enhanced growth of *Arcobacter-*like spp. from marine and shellfish matrices*.* For this purpose, 14 different *Arcobacter*-like spp*.* mostly isolated from shellfish, were grown in 24 different formulations of enrichment broths. The enrichment broths consisted of five main groups based on the organic contents (fresh oyster homogenate, lyophilized oyster either alone or in combination with other standard media), combined with artificial seawater (ASW) or 2.5% NaCl. Optical density (OD_420nm_) measurements after every 24 h were compared with the growth in control media (Arcobacter broth) in parallel. The mean and standard deviation were calculated for each species in each broth and statistical differences (*p* < 0.05) among broths were calculated by ANOVA. The results indicated that shellfish-associated *Arcobacter*-like species growth was significantly higher in Arcobacter broth + 50% ASW and the same media supplemented with lyophilized oysters. This is the first study to have used fresh or lyophilized oyster flesh in the enrichment broth for isolation of shellfish-associated *Arcobacter*-like spp*.*

## Introduction

*Arcobacter*-like species are gram negative, slightly curved, rod-shaped bacteria. The genus *Arcobacter* was separated from the genus Campylobacter in 1991 by Vandamme et al.^1^ and rapidly enlarged with the description of many new species, the majority of them described from shellfish, embracing in 2017 a total of 27 species. The recent taxonomic re-classification grouped these species in seven different genera, leaving only the type species *Arcobacter nitrofigilis* in the original genus^2^. For the purpose of this study, we will mainly either use the original species names or refer to all the species as *Arcobacter*-like species. These species are fastidious organisms and were differentiated from the campylobacters due to their ability for growth in the presence of oxygen and at significantly lower temperatures i.e. 15–30 °C^3,4^.

*Arcobacter*-like species are cosmopolitan in their nature because they are found in different environments, such as water, septic tanks^5,6^, human faeces^7^, sewage, water treatment plants^6,8–13^, food products^9,14^, vegetables and vegetables processing plants^4,15,16^. They are also found in dairy products and buffalo milk^17,18^. They are also isolated from meat in pork and beef^8,19^ slaughterhouses in Portugal^20^. They are commonly associated with seafood^3,21,22^ such as shellfish^23–26^, abalone^27^, lobster^28^, mussels, oysters, clams and seawater^29,30^. They are also reported from zooplankton, such as copepods^31^, as symbionts with other animals, for example as endo-cytobiont of amoeba^32^ and in mutualism with marine animals^33^. They had been sporadically found in association with reptiles^34^ and dogs mouth`s and faeces^35^.

Despite their diversity and isolation from different environments, *Arcobacter*-like species are also considered zoonotic and enteropathogenic, since they have been isolated during several infections and disease episodes both in humans and in animals^36^. Many *Arcobacter*-like species, now included in the new genus *Aliiarcobacter* i.e., *A. butzleri, A. cryaerophilus, A. skirrowii, A. cibarius* and *A. thereius* were considered important pathogens linked to gastrointestinal disease, causing diarrhoea in humans and abortion and enteritis in animals^2,3,21,37–39^. While, *A. butzleri* has been classified as an emerging food pathogen and serious health hazard to humans by International Commission on Microbiological Specifications for Foods (ICMSF)^40^ in 2002. A recent study reported for the first time that the species of marine origin *Arcobacter mytili* (now named *Malaciobacter mytili*) as responsible for a case of bacteraemia^41^, suggesting there exists a virulence potential by these bacteria in humans.

Therefore, water, food products of animal origin and shellfish have been considered as reservoirs and potential transmission routes for potentially pathogenic *Arcobacter*-like species^3,26,42^. The prevalence rate of *Arcobacter*-like spp. from food products, from highest to lowest, is from poultry, seafood, pork meat, dairy products, lamb, and beef followed by rabbit^43,44^.

Despite some authors having underlined the need for developing standardized culture media, until now, no official standard protocol exists for the isolation of *Arcobacter*-like spp. from any specific kind of products or environment^3^. Moreover, a pre-enrichment step is needed to enhance the isolation of *Arcobacter*-like species and therefore isolation is time-consuming, requiring at least 72–96 h for the growth of pure bacterial cultures^30,45^. During isolation of bacteria on synthetic media, some bacteria may undergo a Viable But Non-Culturable (VBNC) state failing to grow, and are therefore not detected due to insufficient nutrients, growth promoting factors, or to other unknown reasons^46^.

Out of 27 *Arcobacter*-like species, 18 have been isolated from marine environments, and 9 of those from shellfish. Regardless, we still lack complete knowledge of the diversity and presence of commensal, as well as pathogenic species found in marine invertebrates^27,30^. The increasing number of *Arcobacter*-like species isolated from shellfish by using different isolation approaches, such as enrichment or addition of salts^27,30^, clearly indicates that there is still great potential for isolation of new *Arcobacter*-like species in nature, and specifically from marine environments. A recent microbiome study of the pacific oyster (*Crassostrea gigas*) demonstrated the presence of *Arcobacter*-like species in relatively higher number along with known oyster pathogens, such as *OsHV-1* and *Vibrio aestuarianus*^47^. This highlights the importance of studying in detail the interaction between the *Arcobacter*-like species and shellfish species and their possible role in the pathogenicity in *C. gigas*^48^.

To be able to isolate *Arcobacter*-like species from different sources efficiently it is crucial to enrich bacteriological culture media by adding specific nutrients in such a way that mimics the original environmental conditions. To date, several enrichment broths and selective media have been used for the isolation and selective culturing from foods. The broth media used are: Arcobacter selective broth (ASB) I^49^, Ellinghausen-McCullough-Johnson-Harris Polysorbate 80 broth (EMJH P80)^50^, Arcobacter enrichment medium (AM)^51^, Johnson and Murano broth (JMB)^52^, ASB II^53^ and Arcobacter broth + 2.5% NaCl^30^. Among the latter media the Arcobacter broth + 2.5% NaCl produced 40% more positive samples and provided a higher diversity of known and new species, 11 and 7 respectively^30^. This remarkable discovery of new species was made possible just by adding NaCl to the standard Arcobacter broth and by sub-culturing on marine agar.

The isolation of several new *Arcobacter*-like species from shellfish increased when new cultivation strategies mimicked the original environmental conditions more closely^29,30,54^. In this work, we are testing the enhanced cultivation of *Arcobacter*-like species associated with shellfish or their environment, by formulating culture media containing shellfish matrices and different percentages of artificial seawater (ASW), with the aim of developing one or more improved enrichment broths to enhance their efficient growth for functional and taxonomic work.

## Results

### Physical appearance of enrichment broth media supplemented with oyster homogenate or lyophilized oyster

The enrichment broth supplemented with oyster homogenate (OH) was of dark green colour and comparatively turbid and more opaque after autoclaving the broth. While enrichment broth media supplemented with lyophilised oyster was transparent like other conventional broths and comparatively pale in colour compared to the control enrichment broth i.e. Arcobacter broth, after autoclaving.

### Evaluation of *Arcobacter*-like species growth in different enrichment broth group

#### Growth of *Arcobacter*-like species in conventional enrichment broth with/or without artificial seawater

**Figure 1:**
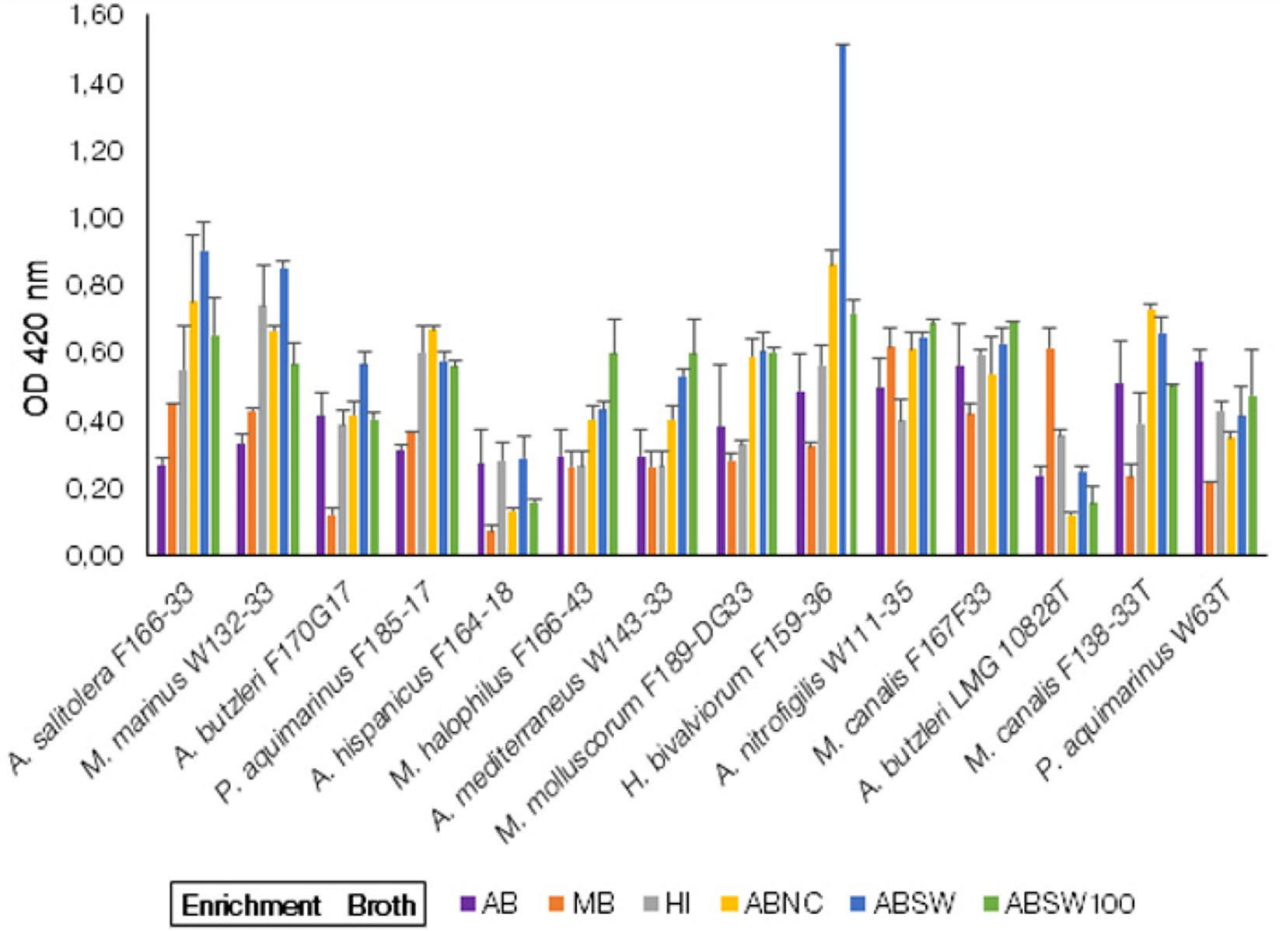
Growth of *Arcobacter*-like spp. in the conventional broth media. AB = Arcobacter Broth (Control Enrichment Broth); MB = Marine Broth; HI = Heart Infusion; ABNC = Arcobacter Broth + 2.5% NaCl; ABSW = Arcobacter Broth + 50% ASW; ABSW100 = Arcobacter Broth + 100% ASW. (Note: The strains with a T in the last are Type strains).

All 14 of the strains tested showed growth in all 6-enrichment broths of this group (Fig. 1) although with different ODs*.* The OD_420nm_ range between 0.4 and 0.8 showed a linear correlation (data not shown), which indicated that the growth of the species reached the exponential phase. A significantly (*p* < *0.05)* higher growth in ABSW was observed for *H. bivalviorum* F159-36 in relation to any other *Arcobacter-*like specie used in this study. The strains of the Candidatus ‘*A. salitolerans’* F166-33, and the strains of the species *M. marinus* W132-33, *A. butzleri* F170G17, and *A. hispanicus* F164-18 also showed a comparatively higher growth in the ABSW enrichment broth than in the control AB broth and other enrichment broth in this group, but these differences were not statistically significant (*p* > 0.05). A higher growth, but without significant difference, was also observed for *M. halophilus* F166-43, *A. mediterraneus* W143-33, and *M. canalis* F167F33 in ABSW100 as compared to AB (Control broth). *A. butzleri* LMG 10828^ T^ showed significantly higher growth in MB (Marine broth). The strains of *P. aquimarinus* F185-17 and *M. canalis* F138-33^ T^ showed significantly higher growth in ABNC compared to control broth (AB). The growth *A. nitrofigilis* W111-35 and *P. aquimarinus* W63^T^ showed uniform growth but lower than 0.4 at OD_420nm_ (No significant difference). The species *A. butzleri* LMG 10828^ T^*, A. hispanicus* F164-18 and *P. aquimarinus* F185-17 showed lower growth at the higher salt content in ASW compared to AB control.

##### Growth of *Arcobacter*-like spp. in oyster homogenate with conventional broth media group

**Figure 2:**
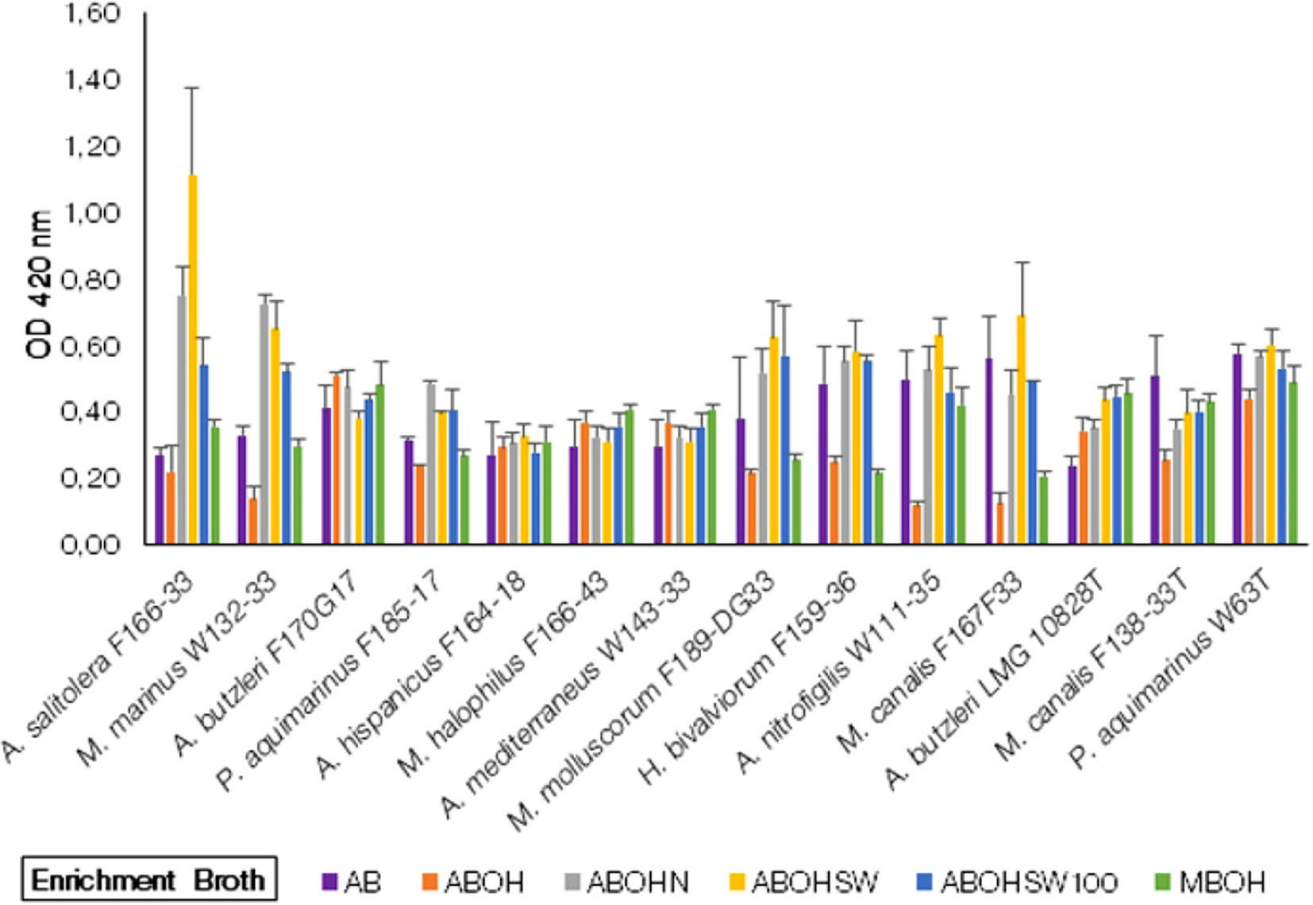
Growth of *Arcobacter*-like spp. in the conventional broth media supplemented with oyster homogenate. AB = Arcobacter Broth (Control Enrichment Broth); ABOH = Arcobacter Broth + Oyster Homogenate; ABOHN = Arcobacter Broth + Oyster Homogenate + 2.5% NaCl; ABOHSW = Arcobacter Broth + Oyster Homogenate + 50% ASW; ABSW100 = Arcobacter Broth + Oyster Homogenate + 100% ASW; MBOH = Marine Broth + Oyster Homogenate. (Note: The strains with a “T” suffix are Type strains).

This group consisted of five different types of broth. The results (Fig. 2) from this media group demonstrated lower growth than from group 1, with an OD_420nm_ below 0.8. The growth of *Arcobacter*-like spp. was comparatively higher in the enrichment broth ABOHSW than in ABOHN, but the difference among the growth was not significant (*p* > 0.05) when compared with the AB control broth media. Candidatus ‘*A. salitolerans’* F166-33, *M. marinus* W132-33*, P. aquimarinus* F185-17*, A. hispanicus* F164-18*, A. mediterraneus* W143-33*, M. molluscorum* F189-DG33*, H. bivalviorum* F159-36*, A. nitrofigilis* W111-35*, M. canalis* F167F33*,* and *A. butzleri* LMG 10828^ T^ showed comparatively higher, though not significant growth, in the control AB media. The growth of all *Arcobacter*-like species was lower in ABOHSW100 enrichment broth except for *A. butzleri* F170G17*, M. halophilus* F166-43*, A. mediterraneus* W143-33 and *A. butzleri* LMG 10828^ T^ which favour higher salinity and have comparatively higher growth in 100% ASW. The growth of most *Arcobacter*-like spp. i.e. Candidatus ‘*A. salitolerans’* F166-33*, M. molluscorum* F189-DG33*, H. bivalviorum* F159-36*, A. nitrofigilis* W111-35*, M. canalis* F167F33*, A. butzleri* LMG 10828^ T^, *M. canalis* F138-33^ T^ and *P. aquimarinus* W63^T^ was higher (no significant difference as *p* > 0.05) in ABOHSW than ABOHN. While *M. marinus* W132-33*, A. butzleri* F170G17*,* and *P. aquimarinus* F185-17 have comparatively higher growth (no significant difference as *p* > 0.05) in ABOHN (Arcobacter broth + 2.5% NaCl supplemented with Oyster homogenate) than ABOHSW. There was no significant difference among all 14 *Arcobacter*-like species when compared with control AB enrichment broth.

##### Growth of *Arcobacter*-like species in oyster homogenate enrichment broth group

**Figure 3:**
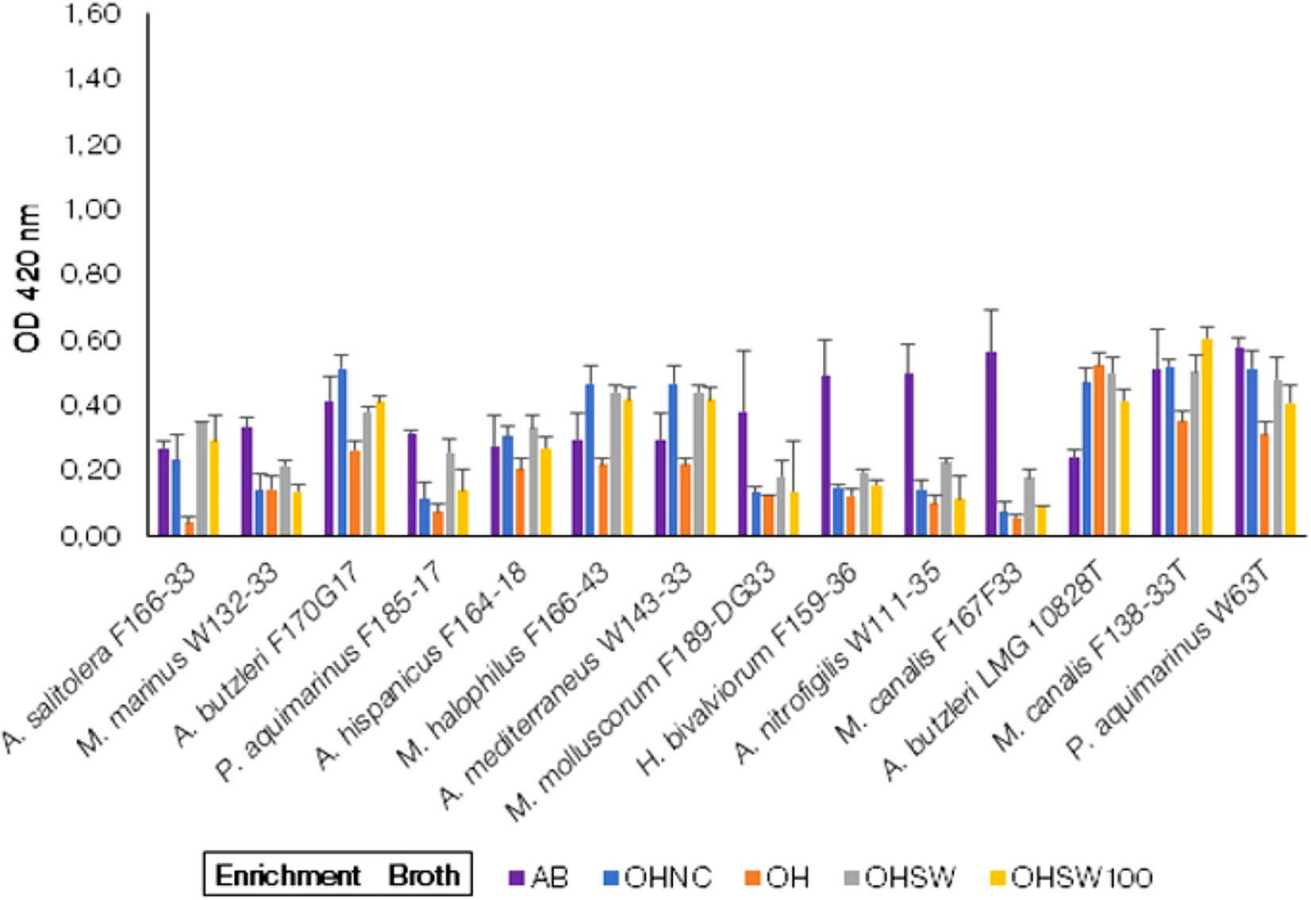
Growth of *Arcobacter*-like spp. in the enrichment broth media prepared from oyster homogenate. AB = Arcobacter Broth (Control Enrichment Broth); OH = Oyster Homogenate + Distilled H_2_O; OHNC = Oyster Homogenate + 2.5% NaCl; OHSW = Oyster Homogenate + 50% ASW; OHSW100 = Oyster Homogenate + 100% ASW. (Note: The strains with a “T” suffix are Type strains).

The enrichment broth group consisted of four enrichment broth media prepared only with oyster homogenate i.e. OH, OHNC, OHSW and OHSW100, without addition of any conventional media components or carbon source. Almost all the strains showed growth (Fig. 3) in these different enrichment broth media but with lower levels than the control broth AB. One exception was for the strain *A. butzleri* LMG 10828^ T^*,* which showed comparatively higher growth than in the control AB broth in comparison to all other strains*,* but the growth was not significantly higher (*p* > 0.05). Many *Arcobacter*-like species i.e. *M. marinus* W132-33, *P. aquimarinus* F185-17, *M. molluscorum* F189-DG33*, H. bivalviorum* F159-36*, A. nitrofigilis* W111-35*,* and *M. canalis* F167F33 showed a higher growth in the control broth AB. The strains of *A. butzleri* F170G17*, A. hispanicus* F164-18*, M. halophilus* F166-43*, A. mediterraneus* W143-33*, A. butzleri* LMG 10828^ T^, *M. canalis* F138-33^ T^ and *P. aquimarinus* W63^T^ could grow in OH with or without salts even in the absence of any additional carbon source from the media components.

##### Growth of *Arcobacter*-like species in conventional broth media supplemented with powder from lyophilized oysters

**Figure 4:**
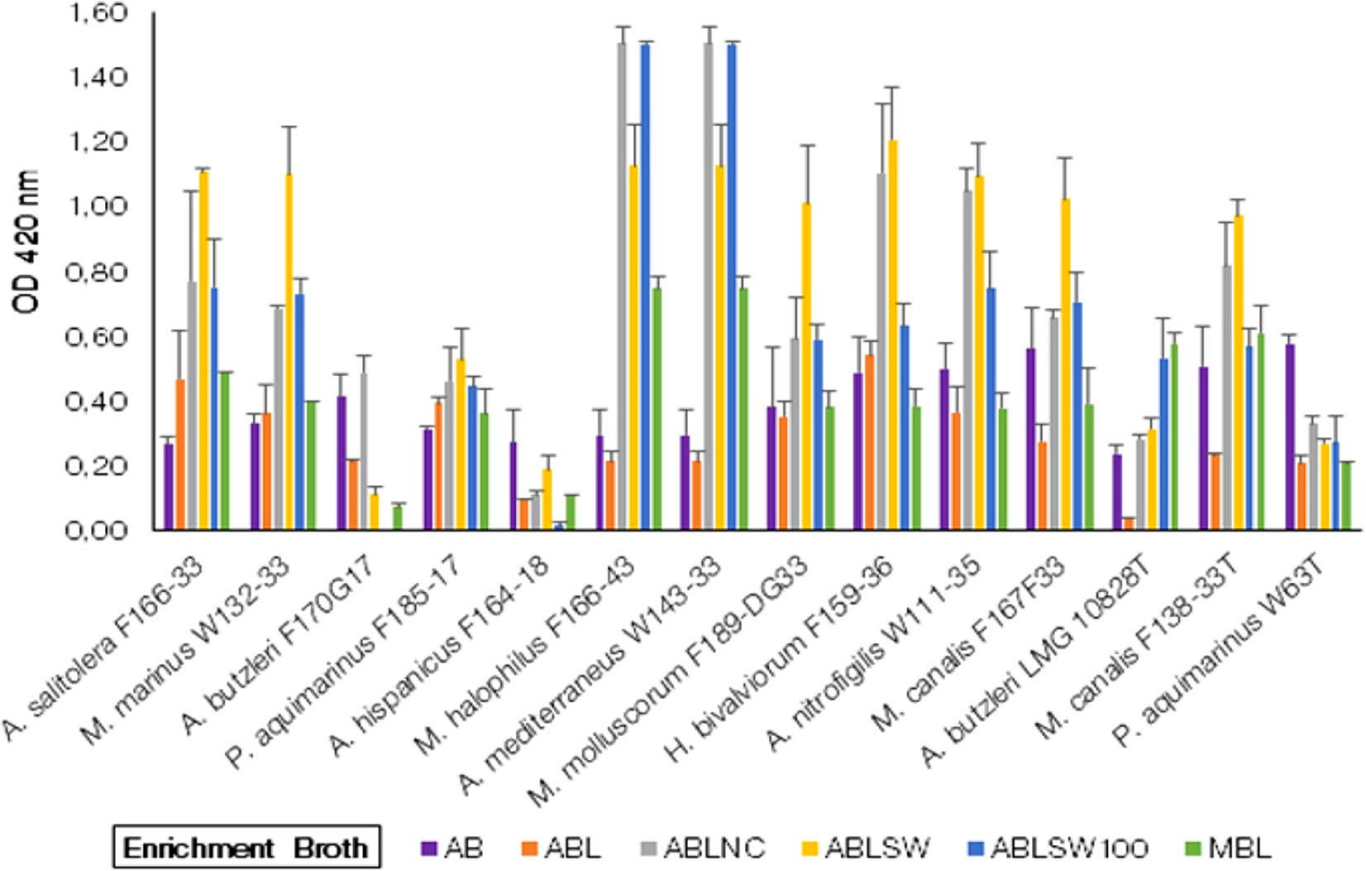
Growth of *Arcobacter*-like spp*.* in conventional broth media supplemented with lyophilized oyster. AB = Arcobacter Broth (Control Enrichment Broth); ABL = Arcobacter Broth + Lyophilized Oyster; ABLNC = Arcobacter Broth + Lyophilized Oyster + 2.5% NaCl; ABLSW = Arcobacter Broth + Lyophilized Oyster + 50% ASW; ABLSW100 = Arcobacter Broth + Lyophilized Oyster + 100% ASW; MBL = Marine Broth + Lyophilized Oyster. (Note: The strains with a “T” suffix are Type strains).

This media group consisted of five types of enrichment broth media prepared by adding to the conventional broth media powder from lyophilized oysters. In this group of enrichment broths ABLSW was the most favourable enrichment broth followed by ABLNC in which almost all *Arcobacter-*like species showed significantly higher growth (*p* < 0.05) compared to the control AB media except for *A. butzleri* F170G17*, A. butzleri* LMG10828^T^, *A. hispanicus* F164-18 and *P. aquimarinus* W63^T^ (Fig. 4). A higher growth (*p* < 0.05) was obtained in ABLSW by Candidatus ‘*A. salitolerans’* F166-33*, M. marinus* W132-33*, P. aquimarinus* F185-17*, M. halophilus* F166-43*, A. mediterraneus* W143-33*, H. bivalviorum* F159-36*, A. nitrofigilis* W111-35*, M. canalis* F167F33 and *M. canalis* F138-33^ T^ compared to the growth in the control AB broth*.* ABLNC was the 2nd most favourable broth media in which Candidatus *‘A. salitolerans’* F166-33*, M. marinus* W132-33*, M. halophilus* F166-43*, A. mediterraneus* W143-33*, H. bivalviorum* F159-36*, A. nitrofigilis* W111-35 and *M. canalis* F138-33^ T^ showed significantly (*p* < *0.05*) higher growth. A significantly higher growth (*p* < 0.05) in ABLSW100 broth media was observed for *M. halophilus* F166-43 and *A. mediterraneus* W143-33 when compared to control AB broth.

##### Growth of *Arcobacter*-like species in salty water or artificial seawater supplemented with powder from lyophilized oysters

**Figure 5:**
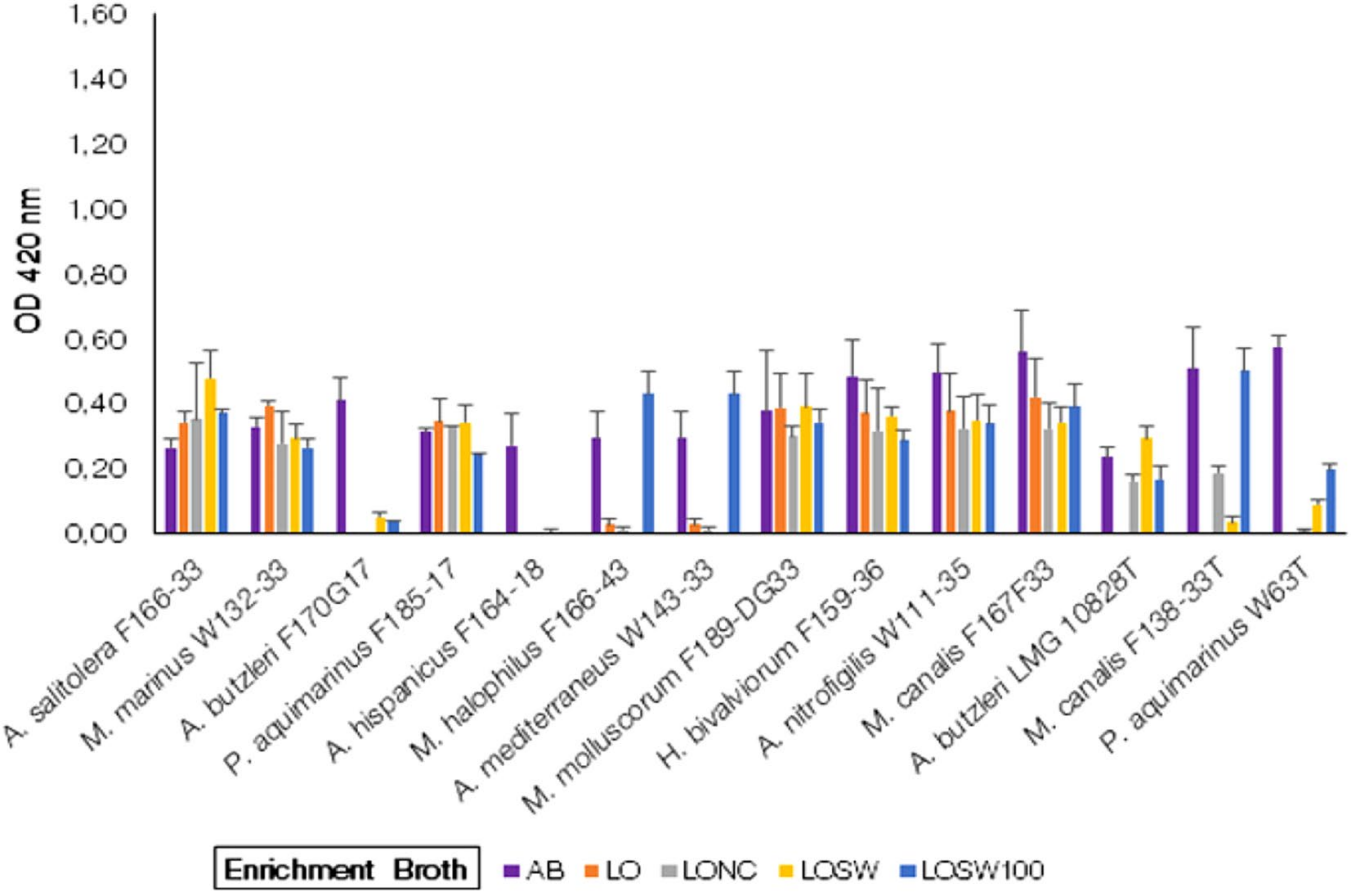
Growth of *Arcobacter*-like spp. in the broth media prepared with lyophilized oyster. AB = Arcobacter Broth (Control Enrichment Broth); LO = Lyophilized Oyster + Distilled H_2_O; LONC = Lyophilized Oyster + 2.5% NaCl; LOSW = Lyophilized Oyster + 50% ASW; LOSW100 = Lyophilized Oyster + 100% ASW. (Note: The strains with a “T” suffix are Type strains).

This group of media were prepared with lyophilized oyster powder in distilled water, alone or with a basal solution of 2.5% NaCl, 50% ASW or 100% ASW. This group consisted of four types of broth media. Growth was observed in almost all four types of broth included in this group for all *Arcobacter-*like species tested (Fig. 5) except *A. butzleri* F170G17*,* and *A. hispanicus* F164-18 which did not grow in any type of broth. The growth was similar or lower than in the control AB broth in all four types of enriched broth media for the strains of the species *M. marinus* W132-33*, P. aquimarinus* F185-17*, M. molluscorum* F189-DG33*, H. bivalviorum* F159-36*, A. nitrofigilis* W111-35*, M. canalis* F167F33*, A. butzleri* LMG 10828^ T^, *M. canalis* F138-33^ T^ and *P. aquimarinus* W63^T^*.* Comparatively higher growth, though not significantly different (*p* > *0.05*), was observed in LOSW for the strain of Candidatus ‘*A. salitolera’* F166-33. The strains *M. halophilus* F166-43 and *A. mediterraneus* W143-33 only grew in LSW100 apart from control AB broth. There were not significant differences (*p* > 0.05) in growth among different species in this media group.

## Discussion

Some *Arcobacter*-like species are considered emerging foodborne pathogens^3^. They were initially reported from terrestrial environments, food and dairy products but many were later on found associated with seafood^9,24,30^. Shellfish have been considered as reservoirs for pathogenic *Arcobacter*-like species for humans^26,44^. These gram-negative rod-shaped bacteria, previously known as the genus *Arcobacter*, have been recently re-classified into seven genera^2^. This new classification is partially supported by the different nutritional requirements for the growth of some of the new genera like the growth in the presence/absence of NaCl^30^. It had been demonstrated that the addition of salt to the culture media favoured the recovery of many new species from shellfish. However, to our knowledge so far it had not been evaluated if the addition of shellfish-based tissues and inter-valval liquid to the enrichment broth could be an organic component that would favour the growth, isolation and recovery of *Arcobacter*-like species from shellfish. For the isolation of bacteria from its natural reservoirs, it is always a strategy by environmental microbiologists to provide, with the best possibility, the same nutrients and environmental conditions found in the sampling site. Microbial interaction among microbial communities in nature and their metabolism is dependent on several parameters like pH, nutrients, osmotic pressure, temperature and several other factors^55^. The failure in isolation of bacteria in many culture media is attributed to inappropriate culture conditions^56^. Therefore, in order to improve isolation of *Arcobacter*-like spp. related to shellfish and/or their environment we aimed to incorporate into the standard Arcobacter broth media, a series of enrichments to better mimic the marine and the shellfish environment. The latter were assayed independently or combined and are the following: seawater (ASW), oyster homogenate (OH), lyophilized oyster (LO) and artificial seawater (ASW). Overall, there were 24 different combinations of enrichment broth, which were divided into five groups of enrichment broth media each with different basal ingredients. The composition of oyster meat is 77–83% moisture content, 9–13% proteins, 1–3% fats, and 1–5% glycogen as a main carbohydrate^57^. According to the work of Zhu et al.^58^, Pacific oyster (*Crassostrea gigas*) contains 79–80% moisture and the composition by percent dry weight was 50–53% proteins, 3–5% fats, 16–22% glycogen and 9–10% ash after freeze drying.

The OH or LO materials were provided in a specific quantity in the enrichment broth in order to get an amount of approximately 0.5% organic content from oyster in combination with conventional enrichment broth (group b and d), or 1% organic contents from oyster (in group c and e enrichment broths). These percentages were calculated based on the organic contents of the conventional broth media i.e. Arcobacter broth, tryptic soy broth, heart infusion and marine broth. From the oyster, organic content 0.5% was used where there was an organic content added from the conventional broth media, while 1% was used in the absence of any organic source other than oyster. The different *Arcobacter-*like species grew in almost all types of broth media, but with large variation between the five groups of enrichment broth. The conventional broth group also comprised three derivatives of Arcobacter broth by adding salts, i.e., Arcobacter broth supplemented with 2.5% NaCl (ABNC), (used previously for isolation of marine *Arcobacter*-like spp^30,59^.) and two concentrations of ASW i.e. 50% and 100% (ABSW and ABSW100 respectively). The growth *Arcobacter*-like spp. was comparatively higher in ABSW than previously used ABNC^30^ except *P. aquimarinus* F185-17 and *M. canalis* F138-33^ T^ where the growth was higher in ABNC.

The major non-essential amino acids were aspartic acid, glutamic acid, arginine, taurine and alanine, which makes about 50% of the total protein content in oyster meat. Leucine, valine, lysine, isoleucine, phenylalanine, methionine and threonine are also present as essential amino acids in the composition of oyster (*C. gigas*) meat. Saturated and unsaturated fatty acids like palmitic acid are also found in *C. gigas* meat. Calcium is the most abundant macro mineral followed in *C. gigas* by Mg along with Zn, Cu, Fe and Se^58^. The results obtained from these experiments shows that *Arcobacter*-like species nutritional requirements vary widely. The overview of results from all the species indicates that seawater has a very positive influence on enhancing growth of *Arcobacter-*like species in the conventional broth media group and in enrichment broth combined with LO (group 3a). The enhanced recovery of *Arcobacter*-like spp*.* by the addition of 2.5% NaCl in Arcobacter broth resulted in the recovery of 40% more *Arcobacter*-like spp.^30^, compared to the previously used Arcobacter broth. Recently, a similar approach used Arcobacter broth + 75% ASW for the isolation of *Arcobacter-*like species from abalone (*Haliotis gigantea*) samples and this resulted in the recovery of two previously unknown *Arcobacter* isolates^27^. The higher growth shown by the species such as Candidatus ‘*A. salitolerans’* F166-33, *M. marinus* W132-33, *M. halophilus* F166-43, *M. molluscorum* F189-DG33, *H. bivalviorum* F159-36 and *M. canalis* F138-33^ T^ in ABSW (group 1) compared to control broth AB, indicates that the growth of these species is significantly influenced by the presence or absence of seawater. These results clearly indicate that using seawater in the media preparation improve their isolation and enhance cultivation of *Arcobacter*-like species from marine environments. In addition to Na and Cl, other ions though collectively in extremely low concentrations, can be critically important for marine organisms^60^. Tyler et al*.*^61^ demonstrated that 35 out of 96 marine bacterial isolates had been isolated from marine samples by adding all 4 major salts i.e. NaCl, MgCl_2_, MgSO_4_, and KCl in 1% (w/v) trypticase broth media. Neither of these isolates could be recovered in the absence of any of these salts. This may be due to the inability of marine bacteria to grow on the substrate or media combination provided as we have very little knowledge of the organic substrate and its concentrations available to those bacteria in the marine environment^62^. Marine bacteria need Na^+^ for their growth, as the ion is necessary for enzyme activity, membrane stability, active transport, respiration-dependent primary Na^+^-pumps and many other functions in bacterial cells^63–67^. The genome analyses of many human and animal bacterial pathogens show that they encode Na^+^ based enzymes i.e. decarboxylases, oxidoreductases and permeases as well as membrane primary Na^+^ pumps instead of, or in addition to H^+^ pumps, and these might be important virulence factors for several pathogenic bacteria^68^. Further, the presence of ions can affect the mechanical properties of biofilms and serves as a cross linker in the attachment during biofilm formation^69,70^. The complete elemental requirements for cell growth (i.e. which elements and in which amounts/ concentration) for *Arcobacter*-like species remains an area for future studies.

Although, the 14 species used in this study (out of 29 *Arcobacter*-like species known) were originally isolated from the marine environment, we still lack complete knowledge on the diversity and presence of commensals or pathogenic species among those genera found in marine invertebrates^27^. The approaches followed by Salas-Masso et al.^30^, Mizutani et al.^27^ and Kim et al.^71^ resulted in enhanced recovery of *Arcobacter*-like species from shellfish, abalone and food samples. These studies indicate that still there is a great potential for isolation of new species, especially from the marine environment.

The supplementation of shellfish tissue (OH and LO) into the enrichment broth significantly enhanced the growth of *Arcobacter*-like species (*p* value < 0.05) especially in the ABLSW enrichment broth (Arcobacter broth + Lyophilized Oysters + 50% ASW) when compared to the control broth AB*.* The supplementation of only LO did not improve the growth in the absence of 50% ASW, which may suggest a synergy between the ASW and the LO supplement for the enhancement of the recovery of strains from marine environments. While the lowest growth was observed in ABOH enrichment broth for most species, the addition of only OH into Arcobacter broth did not have a promising effect on the growth either. The control AB broth when supplemented with LO flesh (i.e. ABL broth) does not seem to improve growth, and even decreased growth for most of the species. However, when ABL was supplemented with salts such as 2.5% NaCl and 50% ASW, there were significant improvements in growth for most of *Arcobacter*-like spp. mentioned above.

Through successive studies, a more efficient broth ASB II (Arcobacter selective broth II) has been adopted for the enrichment of *Arcobacter*-like spp.^72^. However, fastidious microorganisms such as *Gemmata obscuriglobus* and *Gemmata massiliana* have been successfully grown using supplements, such as ground fresh sponge tissues, in culture media^56^. Similarly, the results herein indicate that Arcobacter broth + 50% ASW (ABSW) and Arcobacter broth + Lyophilized Oyster + 50% ASW (ABLSW) are a better enrichment broth for the isolation of *Arcobacter*-like species from shellfish and marine environments, as compared to the Arcobacter broth used in many studies. Therefore, these protocols should be adopted in future studies for the isolation of *Arcobacter*-like species from shellfish samples. Unidentified specific components of oyster flesh/ASW may contain key trace compounds necessary for fulfilling nutritional requirements and enhancing growth of some *Arcobacter*-like spp. The biochemical composition of *C. gigas* flesh is a rich source of proteins, essential and non-essential fatty acids, and macro and micro minerals^58^. Preferably, lyophilized oysters should be used in the media along with ASW, rather than oyster homogenate because this confers to the broth media a dark green colour and increases turbidity thereby obscuring observations.

The results obtained from this study suggest that marine-derived *Arcobacter*-like species have somewhat specific nutritional requirements. The approach used in this study should result in more efficient isolation and cultivation of marine-derived *Arcobacter*-like species in the future and could help enhance the description of diversity of species obtained from shellfish. These finding suggests that Arcobacter broth + 50% artificial seawater (ABSW) is the best enrichment broth medium for the growth of marine based *Arcobacter*-like spp. A more detailed work is required to refine the lyophilized oyster material used for supplementation and study the effect of different biochemical components in the lyophilized oyster on the growth of individual species or on the different genera as target groups.

## Material and methods

### Bacterial isolates

The experiment was performed with 14 different *Arcobacter*-like species that belonged to 5 out of 7 different genera according to the new classification of Perez-Catalunya et al.^2^ shown in Table 1*.* Most bacterial isolates used in this study were previously isolated in IRTA (Sant Carles de la Rapita, Spain) and Universitat Rovira i Virgili (Reus, Spain) from shellfish or their water environment; type strains have been included for comparative purposes (Table 1). Out of 14 *Arcobacter*-like species used in this study, 13 species were isolated previously from Alfacs Bay and shellfish exposed to Poble Nou Channel Water and one *Arcobacter*-like Type strain was purchased from Spanish Culture Collection, CECT (Table 1). The strains were refreshed from − 80 °C glycerol stock suspensions on respective solid media i.e. marine agar and blood agar media and incubated aerobically for 48 h at 30 °C.

**Table 1:** List of *Arcobacter*-like strains (origin, isolation source, first report) used for the development of an improved enrichment broth for the isolation of *Arcobacter*-like spp.

Strain name	Old name	Strain code in URV collection	Enrichment broth	Isolation media	Isolation source	Source of first report of the species
Candidatus *Arcobacter salitolerans*	Candidatus *Arcobacter salitolerans*	F166-33	Arcobacter CAT broth + 2.5% NaCl	Marine Agar	Oyster exposed to Poble Nou Channel Water	Oyster exposed to sewage water^43^
*Aliiarcobacter hispanicus*	*Arcobacter hispanicus*	F164-18	Arcobacter CAT broth	Blood Agar	Oyster (Alfacs Bay)	Oyster Alfacs Bay^2^
*Halarcobacter mediterraneus*	*Arcobacter mediterraneus*	W143-33	Arcobacter CAT broth + 2.5% NaCl	Marine Agar	Poble Nou Channel Water	Mussel Alfacs Bay^2^
*Arcobacter nitrofigilis*	*Arcobacter nitrofigilis*	W111-35	Arcobacter CAT broth + 2.5% NaCl	Marine Agar	Poble Nou Channel Water	Marshland plant roots^73^
*Aliiarcobacter butzleri* (Type)	*Arcobacter butzleri* (Type)	LMG 10828^ T^	Direct cultured on blood agar medium	Blood Agar	Man, with diarrhoea, faeces (USA, 1990)	Human and Animal diarrheal Samples ^74^
*Aliiarcobacter butzleri*	*Arcobacter butzleri*	F170G17	Arcobacter CAT broth	Blood Agar	Oyster exposed to Poble Nou Channel Water	Human and Animal diarrheal Samples^74^
*Halarcobacter bivalviorum*	*Arcobacter bivalviorum*	F159-36	Arcobacter CAT broth + 2.5% NaCl	Marine Agar	Oyster exposed to Poble Nou Channel Water	Mussel from Ebro Delta, Spain^24^
*Pseudarcobacter aquimarinus* (Type)	*Arcobacter aquimarinus* (Type)	W63^T^ = CECT 8442^ T^ = LMG 27923^ T^	Arcobacter CAT broth	Blood Agar	Seawater, Garraf beach, Catalonia, Spain^29^	Seawater, Garraf beach, Catalonia, Spain^29^
*Pseudarcobacter aquimarinus*	*Arcobacter aquimarinus*	F185-17	Arcobacter CAT broth	Blood Agar	Mussel exposed to Poble Nou Channel Water	Seawater, Garraf beach, Catalonia, Spain ^29^
*Malaciobacter molluscorum*	*Arcobacter molluscorum*	F189-DG33	Arcobacter CAT broth + 2.5% NaCl	Marine Agar	Mussel exposed to Poble Nou Channel Water	Mussel, Ebro Delta, Spain^23^
*Malaciobacter canalis* (Type)	*Arcobacter canalis* (Type)	F138-33^ T^ = CECT 8984^ T^ = LMG 29148^ T^	Arcobacter CAT broth + 2.5% NaCl	Marine Agar	Oyster exposed to untreated sewage at Poble Nou Canal, Spain ^2^	Oyster exposed to untreated sewage at Poble Nou Canal, Spain ^2^
*Malaciobacter canalis*	*Arcobacter canalis*	F167F33	Arcobacter CAT broth + 2.5% NaCl	Marine Agar	Alfacs Bay Mussel	Oyster exposed to untreated sewage at Poble Nou Canal, Spain ^2^
*Malaciobacter marinus*	*Arcobacter marinus*	W132-33	Arcobacter CAT broth + 2.5% NaCl	Marine Agar	Alfacs Bay Water	Seawater, Dokdo Island, Korea^75^
*Malaciobacter halophilus*	*Arcobacter halophilus*	F166-43	Arcobacter CAT broth + 2.5% NaCl	Marine Agar	Oyster exposed to Poble Nou Channel Water	Water from Hypersaline lagoon, HawaiianIslands^76^

### Artificial seawater (ASW) preparation

Artificial seawater (ASW) was prepared following the sea salt manufacturer's instructions (Aqua Medic MeerSalz, by AB Aqua Medic GmbH, Germany) by adding 31.4 gm per litre of distilled water, pH 8 ± 0.2, and salinity 34‰. All the media requiring seawater were prepared by using this ASW.

### Growth monitoring

Growth was assessed by optical density (OD) readings at 420 nm, by means of a photometer (Model D-100, Dinko instruments, Spain). A preliminary OD vs. colony forming unit (CFUs) in marine agar, was performed with *M. canalis* to evaluate its correlation. The mean value was calculated considering the CFU counts obtained from triplicates of each plate and of every dilution. An OD vs. CFU correlation was made for all the three OD value (0.4, 0.6 and 0.8) tested.

### Preparation of oyster matrices for media supplementation

Two types of oyster matrices were prepared as the organic nutrient source and were supplemented into the conventional broth media, as explained below.

#### Oyster homogenate (OH)

Two kilograms (2 kg) of depurated oysters (*Crassostrea gigas*) produced locally in the Ebro Delta bays (Tarragona, Spain) were purchased from a commercial store. The oysters (n = 30) were of commercial size, ranging from 65 to 97 cm in shell length. They were rinsed and cleaned externally before opening. The oyster flesh (299 g) with its inter-valval liquid was collected in a sterile beaker. Then the samples were homogenized with a sterile kitchen blender. The homogenized oyster flesh was used in the preparation of the different kinds of broth used in the experiments.

#### Lyophilized oyster (LO)

The Pacific oysters (*C. gigas*) were obtained from Ebro Delta, Spain and dry mass of lyophilized oysters, was prepared by freeze drying flesh and inter-valval liquid of oysters in a lyophiliser. The dry mass was then ground to a fine powder. The fine powder obtained after grinding was used as a supplement in the different experimental broth media.

### Media preparation

#### Broth media preparation

Five groups of different broth media were prepared. These groups were based on using three conventional broth media with or without oyster supplementation (OH or LO) and prepared using different salt compositions (NaCl/ Artificial seawater [ASW]) and strengths (50% ASW or 100% ASW). The pH and salinity of each broth was measured. The experiment was done in duplicate.

Details of the five groups, making 24 types of broth media (Table 2) are given below;

1. **Conventional broth media**: The three conventional broth media used (Arcobacter broth, AB; Marine broth, MB and Heart Infusion, HI) were prepared according to the instructions from the manufacturer (Table 2). The Arcobacter broth was modified by adding 2.5% of NaCl^30^ or by adding 50% and 100% artificial seawater (ASW).
2. **Enrichment broth media containing oyster homogenate**:
  - Conventional broth media supplemented with oyster homogenate (OH): This group of media were prepared by mixing half of the amount specified by the manufacturer instructions for conventional broth media (12g/L of Arcobacter broth _AB (Oxoid, UK) or 18.7 g/L of marine broth _MB (BD, Spain), with 25 g/L of homogenized oyster (HO). Additionally, three types of Arcobacter broth supplemented with homogenized oyster (ABOH) were prepared by adding 2.5% NaCl or 50% ASW or 100% ASW. Thus, this group contained five types of broth media (Broth# 7-11 of Table 2).
  - 2b. Oyster homogenate broth media: This media was prepared by adding 50 g/L of oyster homogenate into distilled water with no salt added or three different concentration (2.5% NaCl or 50% ASW or 100% ASW). It consisted of four types of broth media (Broth# 12-15 of Table 2).
3. **Enrichment broth media containing lyophilized oyster**:
  - Conventional broth media supplemented with lyophilized oyster: This group of media was prepared by supplementing 12 g/L Arcobacter broth or 18.7 g/L marine broth i.e. half amount of that given by manufacturer instruction for both conventional broth media, with 5 g/L lyophilized oyster. Arcobacter broth supplemented with LO (ABLO) were also prepared with 2.5% NaCl or 50% ASW or 100% ASW. This group contained five types of broth media (Broth# 16-20 of Table 2).
  - Lyophilized oyster broth media: This group of media consisted of four types of broth (Broth# 21-24 of Table 2) by adding 10 g/L of lyophilized oyster powder in to either distilled water or distilled water containing 2.5% NaCl or 50% or 100% ASW.

**Table 2:** List of 24 broth media used in the experiment.

Broth media	Broth #	Broth media name	Broth media abbreviation	Salinity (ppt)	pH
Conventional Broth Media	1	Arcobacter Broth	AB	7.6	7.06
	2	Marine Broth	MB	25.3	7.56
	3	Heart Infusion	HI	7.3	7.53
	4	Arcobacter Broth + 2.5% NaCl	ABNC	27.8	7.10
	5	Arcobacter Broth + 50% Artificial Sea Water (ASW)	ABSW	16.9	7.03
	6	Arcobacter Broth + 100% ASW	ABSW100	31.5	7.55
Conventional Broth Media + Homogenized Oyster	7	Arcobacter Broth + Oyster Homogenate (OH)	ABOH	4.2	7.13
	8	Arcobacter Broth + OH + 2.5% NaCl	ABOHN	25.7	7.04
	9	Arcobacter Broth + OH + 50% ASW	ABOHSW	16.8	7.13
	10	Arcobacter Broth + OH + 100% ASW	ABOHSW100	28.6	7.29
	11	Marine Broth + OH	MBOH	25.6	7.41
Homogenized Oyster Media	12	OH + d.H_2_O	OH	1.2	7.09
	13	OH + 2.5% NaCl	OHNC	26.3	6.69
	14	OH + 50% ASW	OHSW	12.6	7.06
	15	OH + 100% ASW	OHSW100	25.8	7.35
Conventional Broth Media + Lyophilized Oyster	16	Arcobacter Broth + Lyophilized Oyster (LO)	ABL	5.6	7.08
	17	Arcobacter Broth + LO + 2.5% NaCl	ABLNC	28.1	6.88
	18	Arcobacter Broth + LO + 50% ASW	ABLSW	16.7	6.98
	19	Arcobacter Broth + LO + 100% ASW	ABLSW100	28.4	7.53
	20	Marine Broth + LO	MBL	25.7	7.56
Lyophilized Oyster Media	21	LO + d.H_2_O	LO	2.2	6.95
	22	LO + d.H_2_O + 2.5% NaCl	LONC	27.1	7.1
	23	LO + 50% ASW	LOSW	14.9	6.93
	24	LO + 100% ASW	LOSW100	27.4	7.25

The abbreviation given in the separate column for each broth media will be used in the figures/tables onwards.

### Experimental procedures

#### Inoculum preparation

Fresh colonies of the 14 *Arcobacter*-like species grown on marine and blood agar mentioned above (Table 1) were transferred to sterile marine broth and tryptic soy broth (TSB) tubes, respectively. The tubes were vortexed for 10 s and incubated in aerobic condition at 30 °C for 24 h to get the uniform fresh culture of the colonies in a liquid medium. The optical density at 420 nm was adjusted, with sterile marine broth, to OD value 0.1 for all the strains used in the study (Table 1)*.*

#### Broth inoculation and incubation:

From the inoculum, 0.1 ml was transferred to the 9.9 ml pre-labelled broth tubes of different enrichment broth, vortexed and incubated in aerobic condition at 30 °C. The OD_420nm_ was measured for each strain at 0 h, 24 h and 48 h in different broth media (Table 2). Sterile broth of each formulation was used as control for the OD reading.

#### Inoculation on solid media:

After the incubation for 48 h, the growth of all the strains in the 24 types of enrichment broth was determined by culturing on marine agar, 100 µl of each enrichment broth. The plates were then incubated in aerobic condition at 30 °C for 48 h. Purity of the cultures was assessed, and relative growth was recorded as number of colonies following a scale from 0 to 4 (0 = No growth; 1 = 1–30 colonies; 2 = 31–150 colonies; 3 = 151–300 colonies; 4 =  > 300 colonies).

### Statistical analysis

The growth results are expressed in the terms of optical density (OD_420nm_) for each strain tested in each enrichment broth used in this study. Values of the mean and standard deviation were calculated for duplicate OD_420nm_ values. Statistical analysis was performed in SPSS 21.0 (IBM SPSS Statistics, SPSS Inc., USA). Analysis of variance (ANOVA) and differences among the mean values were tested by Tukey's test. Significant difference was accepted for *p* < 0.05.
